# Chronic traumatic encephalopathy and other neurodegenerative proteinopathies

**DOI:** 10.3389/fnhum.2014.00030

**Published:** 2014-01-31

**Authors:** Maria Carmela Tartaglia, Lili-Naz Hazrati, Karen D. Davis, Robin E. A. Green, Richard Wennberg, David Mikulis, Leo J. Ezerins, Michelle Keightley, Charles Tator

**Affiliations:** ^1^Division of Neurology, Krembil Neuroscience Centre, University Health Network, University of TorontoToronto, ON, Canada; ^2^Tanz Centre for Research in Neurodegenerative Disease, University of TorontoToronto, ON, Canada; ^3^Canadian Sports Concussion ProjectToronto, ON, Canada; ^4^Department of Laboratory Medicine and Pathobiology, University of TorontoToronto, ON, Canada; ^5^Division of Neurosurgery, University Health Network, University of TorontoToronto, ON, Canada; ^6^Division of Brain, Imaging and Behaviour – Systems Neuroscience, Toronto Western Research Institute, University Health NetworkToronto, ON, Canada; ^7^Department of Surgery, University of TorontoToronto, ON, Canada; ^8^Institute of Medical Science, University of TorontoToronto, ON, Canada; ^9^Toronto Rehabilitation InstituteToronto, ON, Canada; ^10^Division of Neuroradiology, Joint Department of Medical Imaging, Toronto Western Hospital, The University of TorontoToronto, ON, Canada; ^11^Executive Director, Canadian Football League Alumni AssociationToronto, ON, Canada; ^12^Bloorview Research Institute, Holland Bloorview Kids Rehabilitation HospitalToronto, ON, Canada; ^13^Department of Occupational Science and Occupational Therapy, University of TorontoToronto, ON, Canada; ^14^Graduate Department of Rehabilitation Science, University of TorontoToronto, ON, Canada; ^15^Department of Psychology, University of TorontoToronto, ON, Canada; ^16^Division of Neurosurgery, Krembil Neuroscience Centre, Toronto Western Hospital, University of TorontoToronto, ON, Canada

**Keywords:** concussions, chronic traumatic encephalopathy, neurodegenerative disease, Alzheimer's disease, frontotemporal lobar degeneration, tau

## Abstract

“Chronic traumatic encephalopathy” (CTE) is described as a slowly progressive neurodegenerative disease believed to result from multiple concussions. Traditionally, concussions were considered benign events and although most people recover fully, about 10% develop a post-concussive syndrome with persisting neurological, cognitive and neuropsychiatric symptoms. CTE was once thought to be unique to boxers, but it has now been observed in many different athletes having suffered multiple concussions as well as in military personal after repeated blast injuries. Much remains unknown about the development of CTE but its pathological substrate is usually tau, similar to that seen in Alzheimer's disease (AD) and frontotemporal lobar degeneration (FTLD). The aim of this “perspective” is to compare and contrast clinical and pathological CTE with the other neurodegenerative proteinopathies and highlight that there is an urgent need for understanding the relationship between concussion and the development of CTE as it may provide a window into the development of a proteinopathy and thus new avenues for treatment.

## Concussions: an evolution in our understanding

“Chronic traumatic encephalopathy” (CTE) is described as a slowly progressive neurodegenerative disease with pathological tau accumulation at the depths of the sulci in superficial layers of the cortex. Clinically CTE is believed to include neuropsychiatric, cognitive and motor deficits that manifest years after implicated concussive or subconcussive events (McKee et al., 2009). It is believed to be a consequence of repeated mild brain traumas also known as concussions. Although the majority of concussions are fully resolved within 3 months (Iverson, 2007), and conventional neuroimaging and neuropsychological testing are typically normal (Broglio and Puetz, 2008), the notion of concussion as a completely benign event (e.g., “bell-ringer”) is outdated. Most people return to baseline after a single concussion but an estimated 10% of cases result in serious and persisting somatic, affective, cognitive, and/or movement sequelae (Wood, 2004). There is growing clinical and societal concern about the effects of multiple concussions (Jordan, 2013) although one meta-analyses revealed no significant effects after multiple concussions (Belanger et al., 2010). CTE was first described in Miller (1966) as a constellation of symptoms typical of neurodegenerative disease. Clinically and pathologically it bears resemblance to other neurodegenerative diseases, now thought of as proteinopathies, which includes Alzheimer's disease (AD) and frontotemporal lobar degeneration (FTLD). Although widespread media attention has spawned a dogma on the *delayed* effects of multiple concussions (Corsellis and Brierley, 1959; Corsellis et al., 1973; Omalu et al., 2005, 2010a; Gavett et al., 2010; Costanza et al., 2011; Stern et al., 2011; Goldstein et al., 2012; McKee et al., 2013), there remains much to be known on the clinical and pathophysiology of CTE. A better understanding of the differences and similarities of CTE and the other proteinopathies may help guide future studies.

## Multiple concussions and chronic traumatic encephalopathy

### Punch drunk syndrome, dementia pugilistica, chronic traumatic encephalopathy

In 1928, Martland introduced the term “punch-drunk” state (Martland, 1928) in reference to the chronic motor and psychiatric consequences of blows to the head in boxing. Millspaugh (1937) coined “dementia pugilistica” to describe similar cases. A few decades later, Critchley (1957) reported on 69 cases of progressive neurological disease in boxers and proposed “chronic progressive traumatic encephalopathy of boxers.” He described an insidious and gradual development of mental and physical anomalies marked by a “euphoric dementia” with emotional lability, little insight, progressive bradyphrenia, and memory deficits, along with changes in behavior. Critchley added that many patients displayed mood-swings, intense irritability, and occasionally, uninhibited violent behavior. He noted “fatuous cheerfulness” as the commonest mood finding but also reported paranoid depression. Motor findings included pyramidal, extra-pyramidal, and cerebellar signs, with tremor and dysarthria the most frequently reported. Sensory perceptual findings included deafness and poor vision. His patients also complained of persistent dull headaches, postural dizziness, and unsteady gait, reminiscent of acute concussion and post-concussive syndrome. In 1969, Roberts reported on 224 former boxers and found that 17% suffered from significant memory loss, aggression, confusion, or depression and that there was direct correlation of incidence to number of fights and overall length of boxing career (Roberts, 1969). Many observational studies, some prospective, have also been undertaken, including a systematic review of 36 of an initial 943 studies on the chronic effects of amateur boxing (Loosemore et al., 2007).

The early literature on the chronic effects of multiple concussions focused on boxing, but multiple concussions sustained under different circumstances can also produce chronic effects. The term CTE has been coined to encompass progressive neurodegenerative effects observed after multiple concussions sustained in any context (Miller, 1966).

Clinical CTE cases overlap with punch-drunk syndrome. CTE is usually described as an evolving constellation of cognitive, psychiatric and motor symptoms (McKee et al., 2009). Cognitive findings may precede, co-occur or follow psychiatric findings, and can include impaired concentration, attention, and memory along with disorientation, confusion, and speech abnormalities later on McKee et al. (2009). Emotional lability, inappropriate behavior, paranoia, outbursts of aggressive behavior and explosivity, mood disturbance, disinhibition, psychosis, and dysexecutive symptoms are observed. Dizziness and headaches are frequent (McKee et al., 2009). Psychiatric symptoms are observable at all stages of CTE, with no clear dose response between extent of neuropathology and clinical symptoms (McKee et al., 2013). Parkinsonian symptoms of tremor, masked facies, wide based gait, poor speech, ocular abnormalities, bradykinesia, and dementia appear as the disease progresses (Omalu et al., 2011a; McKee et al., 2013).

### Who is at risk?

The majority of cases of suspected CTE have been reported in athletes in contact sports, including boxing, hockey, wrestling, soccer, and North American football (Corsellis et al., 1973; Omalu et al., 2006, 2010b; McKee et al., 2009; Dekosky et al., 2010; Gavett et al., 2011; Neselius et al., 2012). CTE has also been associated with physical abuse and epilepsy (McKee et al., 2009). More recently, CTE was reported in a war veteran having suffered blast-injury without signs of overt concussion (Omalu et al., 2011b).

CTE requires post-mortem assessment and all post-mortem studies to date contain samples that are not representative of either the general population or even of multiply concussed populations. McKee et al. (2009) reported that 46/51 (90%) of neuropathologically confirmed CTE cases occurred in athletes who had played contact sports. However, in general, brains referred for autopsy are those of individuals who displayed overt neurological signs at the time of death and therefore were at elevated risk of underlying pathology compared to the large number of athletes who play contact sports but do not show neurological signs throughout their life.

McKee et al. (2013) recently published an expansion of her previous study with 85 brains from former athletes, veterans and civilians with a history of multiple concussions. Importantly, there was no evidence of CTE despite repetitive concussion in 17/85 (20%) of cases, and in 15/85 cases (37%) there was significant comorbid pathology of AD, Lewy body disease (LBD), motor neuron disease (MND), or FTLD. In advanced cases, a comorbid condition such as AD, LBD, or FTLD was present in almost half the cases (10/25) These findings converge with a recent study undertaken by our group, which showed that even with a history of multiple concussions from contact sport *and* a positive clinical presentation before death, a diagnosis of CTE is not inevitable on neuropathological examination. Our case series included the brains of six retired professional players of the Canadian Football League with a history of multiple concussions (Hazrati et al., 2013) and all clinically symptomatic before death. While each case displayed significant neuropathological changes on post-mortem examination, only three showed pathology consistent with CTE. In the other three cases, the neuropathological diagnoses were Parkinson's Disease (PD), Amyotrophic Lateral Sclerosis (ALS), and AD. Even the cases with CTE had co-pathology, including AD, PD, or vasculopathy. This study demonstrates that there is not always a direct relationship between multiple concussions, clinical symptomology, and CTE.

In a recent retrospective analysis a higher incidence of mortality from neurodegenerative disease including AD- and ALS was reported among former National Football League players, compared to the general population (Lehman et al., 2012). Notably, however, players (vs. the rest of the general US population studied), and in particular players in speed positions, showed the *lowest* death rate for other causes of death including cardiovascular disease and cancer, the largest killers of the general US population. These data may thus inflate the apparent risk of neurodegenerative disease and argue for prospective research into the biological effects of multiple concussions.

### Age of onset

Critchley (1957) reports age of onset on 11 cases of punch-drunk syndrome: two were still boxing, six were in their 20 s, two were in their 30 s, and one was 61 years old. McKee et al. (2009) reported CTE symptom onset at ages ranging from 25 to 76 years; one-third were symptomatic at retirement and half were symptomatic within 4 years of their retirement. Although Omalu et al. (2011a) proposed an asymptomatic period between playing of the sport and symptom onset, in his series of 10 cases, four were in their 30 s, three were in their 40 s, and three were in their 50 s. Thus, very few of the players had a prolonged asymptomatic or latent period. There has been no differentiation between the delayed onset of CTE-associated symptoms and the more immediate onset observed in boxers. Importantly, in the absence of serial, longitudinal evaluations in the above studies, we cannot rule out symptom onset prior to formal diagnosis.

Whether CTE and the punch-drunk syndrome are dissociable entities is still unclear. While both syndromes can be associated with the cumulative effects of concussion, it is conceivable that CTE reflects a delayed onset entity, while the punch-drunk syndrome represents a continuation and progression of symptoms from an acute concussive state (Gardner et al., 2014). Also uncertain is whether some players are in a prolonged or more severe postconcussive syndrome that may have a different pathophysiology than the players who develop symptoms decades after their last concussion.

## Chronic traumatic encephalopathy vs. other proteinopathies

### Clinical comparisons

The clinical diagnosis of CTE is currently not feasible due to the overlap with other neurodegenerative conditions. AD is the most common neurodegenerative disease in those over age 65 (Prince et al., 2013) and most often presents with impaired learning and recall of recently learned information (McKhann et al., 2011). Neuropsychiatric symptoms including depression, apathy, agitation, and irritability, as in CTE, are not uncommon (Cummings, 1997).

**Figure 1:**
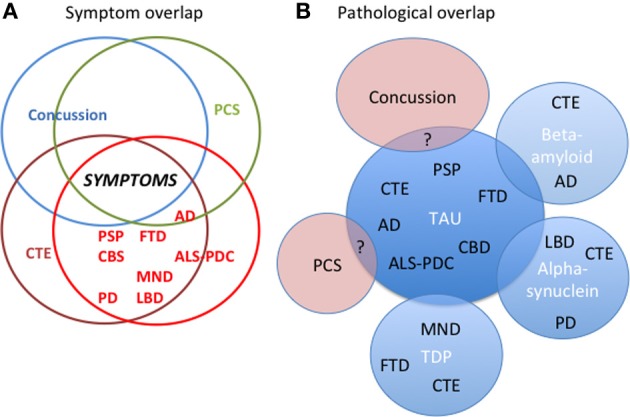
**The interrelationships between concussion, post-concussion syndrome (PCS), chronic traumatic encephalopathy (CTE), and all the neurodegenerative diseases. (A)** There is considerable symptom overlap between concussion, PCS, CTE, and the neurodegenerative diseases. **(B)** Pathologically the relationship between concussion, PCS, CTE, and all the neurodegenerative diseases is unclear. In CTE there is substantial evidence for overlapping pathology of tau, TDP-43, amyloid, and alpha-synuclein with neurodegenerative diseases. In neurodegenerative diseases such as AD, PD, and LBD, there is also overlapping pathology. The pathology of concussion and PCS and their relationship to CTE remains to be explored. AD, Alzheimer's Disease; ALS-PDC, amyotrophic lateral sclerosis-parkinson's dementia complex (Guam); CBD, corticobasal degeneration; CBS, corticobasal syndrome; CTE, chronic traumatic encephalopathy; LBD, Lewy Body Disease; FTD, frontotemporal dementia; MND, motor neuron disease; PCS, Post-concussive syndrome; PSP, progressive supranuclear palsy; PD, Parkinson's disease. “?” indicates uncertainty about overlap.

FTLD is the most common neurodegenerative disease in those less than 65, and includes several clinical syndromes involving changes in behavior, language, and motor function. The main clinical phenotypes are: behavioral variant frontotemporal dementia (bvFTD), primary progressive aphasia, FTD-motor neuron disease (FTD-MND), progressive supranuclear palsy (PSP), and corticobasal syndrome (CBS). Although these syndromes strongly overlap in clinical, genetic, and pathological features their syndrome specific clinical expression differs markedly due to focal pathology (Brun, 1993) (Figure 1A). bvFTD, as its name implies, is primarily a behavioral syndrome characterized by dramatic personality and behavioral changes. The apathy, loss of social norms, and decreased empathy seen in bvFTD are frequent symptoms in traumatic brain injury (TBI) including moderate-severe TBI, concussion, and CTE and are attributed to frontal lobe degeneration (Damasio et al., 1991; Stuss et al., 2001; Jordan, 2013) in particular orbitofrontal and ventromedial prefrontal cortex, as well as insula (Rosen et al., 2005). The language variants, as well as CBS (a progressive, asymmetric, akinetic-rigid syndrome) and PSP (cognitive and behavioral deficits and prominent oculomotor and movement impairments) (Litvan et al., 1996) have less in common with CTE. bvFTD-like symptoms can co-occur in patients with MND (Lomen-Hoerth et al., 2003), and some patients with CTE have presented with MND phenotype and a TAR-DNA binding protein 43 (TDP-43) pathology (McKee et al., 2010). Parkinsonism and cognitive deficits as seen in LBD and PD are also seen in CTE and there are cases of LBD and PD subsequent to multiple concussions (Hazrati et al., 2013; McKee et al., 2013).

The overlap in signs and symptoms of CTE and other neurodegenerative diseases, especially bvFTD, is likely a result of focal involvement of frontal networks that subserve numerous higher functions including personality/emotional life, executive function, and motivation (Stuss and Levine, 2002). A better understanding of the pathophysiology of any of the neurodegenerative diseases may shed light on the risk factors for developing CTE.

### Pathological signs of CTE vs. other neurodegenerative disease

Only a limited number of cases of suspected CTE have undergone pathological examination but post-mortem assessments show a distinct pattern from other proteinopathies including dilated ventricles, a fenestrated, and cavum septum pellucidum, significant atrophy of the medial temporal lobes, thalamus, and mammillary bodies and occasionally pallor of the locus coeruleus and substantia nigra (McKee et al., 2009; Gavett et al., 2011; Stern et al., 2011). Hyperphosphorylated tau deposits in neurons of specific areas of the brains of boxers and professional football players, thus adding CTE to the list of known tauopathies that includes PSP, CBD, FTLD, Guam-Parkinson Dementia Complex, and AD. Tau is a protein that binds to and stabilizes microtubules required for maintaining neuronal shape and for transport of cellular cargo (Brunden et al., 2009). The distribution of these pathological changes is along the amygdalo-hippocampal-septo-hypothalamic-mesencephalic continuum, which is part of the emotional or visceral brain (McKee et al., 2009). The pathological changes are greatest in the depths of sulci, perivascularly around small vessels, and in the superficial cortical layers (II/III). This tau distribution pattern is distinct from other tauopathies. In AD, the neurofibrillary tangles (NFTs) are more regular, are primarily in layers III and V of the cortex, and are neither perivascular nor primarily at the depths of the sulci. In PSP, the NFTs are mainly in the basal ganglia and hindbrain structures. Other distinguishing patterns of tau deposition seen in CTE include irregular and patchy distributions and also prominence in the periventricular and subpial areas. Unlike AD, CTE usually lacks significant amounts of beta-amyloid plaque deposition (Braak and Braak, 1997; McKee et al., 2009; Gavett et al., 2011; Stern et al., 2011).

In select cases of CTE, widespread TDP-43 immunopositive inclusions have been observed (McKee et al., 2010). Lesions involving the corticospinal tracts and the anterior horns of the spinal cord are associated with clinical motor findings of spasticity, weakness, and fasciculations similar to those seen in ALS (McKee et al., 2010). The pathological accumulation of TDP-43 is also seen in FTLD and like tau can cause various FTLD syndromes (Whitwell and Josephs, 2011). Alpha-synuclein positive Lewy bodies as seen in PD and LBD and Alzheimer's beta-amyloid pathology has also occasionally been reported in CTE (McKee et al., 2013).

Similarities exist between CTE and the chronic effects of moderate and severe TBI, in which there can be neurodegeneration in the chronic phase months to years after TBI with sub-acute atrophy within the hippocampi (Ng et al., 2008) and elsewhere (Green et al., 2010). Interestingly, the corpus callosum (unmyelinated axons in particular) is vulnerable to protein deposition post-TBI, suggesting commonality with CTE (Reeves et al., 2005, 2012).

Recently Omalu et al. (2011a) proposed four histomorphologic phenotypes in CTE based on the distribution of NFTs and neuritic threads in the cortex, brainstem, subcortical nuclei, basal ganglia, and cerebellum, and they include amyloid plaques in one phenotype. In contrast, McKee et al. (2013) proposed a staging scheme for CTE severity based on tau distribution which would range from focal epicentres of phosphorylated tau (p-tau) usually in the frontal cortex and typically around small vessels at the depths of sulci to widespread p-tau pathology in a patchy irregular distribution in cortical areas and medial temporal lobe as well as in thalamus, hypothalamus, mammillary bodies, basal ganglia, brainstem and in white matter tracts. Currently, the clinical-pathological relationship is unknown and questions as to whether differences relate to different types of CTE, different types of injury and/or different clinical syndromes remain. There is some evidence for pathological and clinical differences between the classic CTE cases and the “modern” form described in the last few years (Gardner et al., 2014). These authors argue that the classic form of CTE does not appear to advance in a predictable and sequential series of stages, and progression of physical symptoms is only present in approximately one-third of cases. Clearly long-term, prospective clinical studies followed by detailed neuropathological examination are needed to help unravel this issue.

It has become increasingly apparent that CTE frequently coexists with other pathologies. In our series of six cases, the three patients with CTE also exhibited other neurodegenerative pathology as noted above (Hazrati et al., 2013). McKee's recent case series (McKee et al., 2013) found co-pathology of CTE with AD, LBD or both in 17 cases and CTE and MND in eight cases. The relative contribution of the different pathological substrates to the clinical symptoms is currently unknown and requires further study. A recent review of the contemporary cases in the literature found that over 50% had copathology with CTE and only 20% had pure CTE (Gardner et al., 2014).

*In vivo* diagnosis of the specific proteinopathy responsible for a neurodegenerative disease is now the goal. Currently, amyloid imaging and cerebrospinal fluid (CSF) biomarkers of amyloid and tau for the *in vivo* diagnosis of AD are available although not in clinical use (Sperling and Johnson, 2013). Regarding the other neurodegenerative diseases, neither imaging nor fluid biomarkers are available for their diagnosis although there are some experimental data coming out in PD (Parnetti et al., 2013; Schapira, 2013) and FTLD (Hu et al., 2010, 2013). In CTE, concussion, and post-concussion syndrome there are a few studies suggesting abnormalities including elevated levels of CSF tau (Neselius et al., 2012; Shenton et al., 2012; Zetterberg et al., 2013) but these lack pathological confirmation and haven't been reproduced. CTE is still in its infancy with regard to defining the clinical syndrome and determining *in vivo* biomarkers of the underlying pathology.

## Summary and conclusions: much remains to be known

Tau deposition and pathological changes in a particular distribution have been observed in cases of multiple concussions. The evidence to date concerning CTE, its association with multiple concussions, and its clinical signs and symptoms comes from case reports, cases series, and retrospective analyses (Graves et al., 1990; Schofield et al., 1997; Mehta et al., 1999; McKee et al., 2013). The symptoms described in CTE overlap with those described in concussion, PCS and the neurodegenerative diseases. Figure 1A. There is a selection bias for many of the reported cases, some died from violent deaths such as suicide or drug overdose and/or were otherwise clinically symptomatic with cognitive symptoms. There are now an increasing number of reports of cases with multiple concussions but no evidence of CTE at autopsy (Hazrati et al., 2013; McKee et al., 2013). The exact relationship between multiple concussions and CTE is ambiguous. Moreover, one must distinguish clinically and pathologically between static, non-progressive cumulative effects of multiple concussions vs. progressive findings of symptomatic neurodegenerative disease. Complicating the situation are cases of a single, but more serious TBI associated with increased risk of dementia (Blennow et al., 2012; Sayed et al., 2013) as well as atrophy and loss of white matter integrity in the sub-acute and chronic stages of injury (Greenberg et al., 2008; Ng et al., 2008; Whitwell and Josephs, 2011; Adnan et al., 2013)

Prospective, longitudinal studies with neuropathological analysis that sample a broader cross section of individuals, including those with a history of multiple concussions but without positive clinical neurological findings prior to death are critically needed. Understanding the relationship of multiple concussions to CTE as well as possible modifiers is paramount for preventing or ameliorating this illness and for finding a cure. Furthermore, and most importantly, by evoking a diagnosis of CTE as a cause of the symptoms and signs and symptoms in multiple concussions, and failing to address treatable and potentially reversible causes of the suffering is a disservice to the patient and a lost opportunity to understand their sequelae (Wortzel et al., 2013).

CTE now joins the family of tauopathies that includes PSP, bvFTD, and AD but there a number of cases of associated TDP and amyloid pathology, which requires further study and clinical correlate (Figure 1B). As well, the pathological relationship of CTE with concussion and post-concussion syndrome remains to be explored. As we move toward protein specific treatments, *in vivo* diagnosis of CTE at an early stage will be imperative for implementing appropriate treatments and to delay, halt, or reverse its progression. In order to do this, good clinical-pathological studies will be required and appropriate biomarkers will have to be developed.

### Conflict of interest statement

The authors declare that the research was conducted in the absence of any commercial or financial relationships that could be construed as a potential conflict of interest.
